# Pulmonary Function after Adenotonsillectomy

**Published:** 2016-11

**Authors:** Mehrdad Rogha, Jaleh Amini, Mostafa Raisi

**Affiliations:** 1 ***Department of Otorhinolaryngology, Isfahan University of Medical Science, Isfahan. Iran.***; 2 ***Poursina Hakim Research Institute, Isfahan University of Medical Science, Isfahan, Iran.***

**Keywords:** Adenotonsillar hypertrophy, Adenoidectomy, Pulmonary function test, Upper airway obstruction

## Abstract

**Introduction:**

Adenotonsillar hypertrophy is a common disorder among children which, without proper treatment, may lead to considerable problems. Although the consequences of this disorder have been studied in other articles, we decided to evaluate the changes in pulmonary function tests in these children after adenotonsillectomy, and the correlation between clinical and spirometric parameters.

**Materials and Methods:**

We conducted a before- and after- clinical trial. Forty children (17 females and 23 males) with a diagnosis of upper airway obstruction due to adenotonsillar hypertrophy were enrolled in this study. Mean age of the participants was 6.9±1.9 years. Eight spirometric parameters were selected for evaluation pre-operatively and 40 days postoperatively. Besides, symptom scores were defined for each patient to assess their disease severity, pre- and postoperatively. Data were analyzed statistically.

**Results:**

Forced vital capacity (FVC) increased from 1.28±0.26% pre-operatively to 1.33±0.24%postoperatively (P=0.05). Peak expiratory flow increased from 2.74±0.65% pre-operatively to 2.84±0.51% postoperatively (P=0.02) and mid expiratory forced expiratory flow (FEF_25–75_) was 1.81±0.48% pre-operatively, increasing to 1.91±0.50% postoperatively (P=0.02). Maximal expiratory flow at 25% of FVC (MEF_25_) increased from 1.09±0.36% pre-operatively to 1.21±0.34% postoperatively (P=0.02). There was no correlation among the other spirometric parameters (FEV_1_, FEV_1_/FVC, MEF_50_ and MEF_75_) pre- and post-operatively (P>0.05). Despite some improvements in pulmonary function indices, there was no correlation between changes in spirometric parameters and severity of the snoring (P>0.05).

**Conclusion:**

Although our findings reveal that adenotonsillectomy had a positive effect on pulmonary function tests, we found no significant correlation between alterations in spirometric parameters and severity of snoring. However, performing a spirometric examination in children with adenotonsillar hypertrophy may be beneficial for assessing the pulmonary status of the affected patient.

## Introduction

Adenotonsillar hypertrophy (ATH) is a common disorder in children (1,2), and the most common cause of upper airway obstruction in this age group (3). The role of ATH as an important etiology in chronic airway obstruction and ventilatory impairment during sleep has been established (4,5). Without the proper treatment, ATH might lead to considerable problems, such as behavioral, cognitive, and cardiovascular concerns (6-10). Disturbed inspiration in ATH may present with hypoxia, hypercarbia, and elevated bicarbonate levels (11), as well as upper airway obstruction and resultant mouth breathing, often leading to pulmonary morbidities (12).

Fortunately, adenotonsillectomy results in a noticeable improvement in quality of life, cognition, and behavior of patients (13,14), but it is still unclear whether it can affect pulmonary function. Till now, few studies have evaluated the effect of ATH and adenotonsillectomy on pulmonary function tests (PFT).

PFTs in children with ATH were examined by Maurizi et al, who concluded that 52% of patients showed parameters indicating lower airway obstruction (15). An experimental study reported by Di Martino et al. revealed that adenoids may have an impact on expiratory flow (16).

Identification of upper airway obstruction using a peak flowmeter and spirometer has been proposed by some experts, with significant changes between pre- and post-surgery spirometric parameters reported (3,12,15,17). Most investigators have suggested that changes in pulmonary function are transient and disappear after treatment (18). Based on our research, no studies have been published on the correlation between clinical and spirometric parameters.

Thus, we conducted this study in order to evaluate PFT in children affected by symptomatic upper airway obstruction due to ATH, and compare pre-and post-operative values to evaluate any correlation between clinical and spirometric parameters.

## Materials and Methods

In a prospective trial conducted from September 2015 to April 2016 in the department of Otorhinolaryngology and Pulmonology of AL Zahra Hospital, Isfahan, Iran, 40 boys and girls (5–14 years of age) were recruited from patients waiting for an adenotonsillectomy procedure. All cases presented with enlarged tonsils graded >2+/ (degrees III and IV), according to the Brodsky classification and a lateral head X-ray proving significant adenoid hypertrophy with an adenoid-nasopharyngeal (A/N) ratio>0.70 according to the Fujioka method (19,20). Cases excluded from the group were those with cardio- pulmonary disease, neurological involvement, obese children (body mass index [BMI] >30kg/m^2^), those with no surgical indication, and those with other causes of nasal obstruction such as polyps, nasal septal deviations, and thoracic skeletal deformity, for example.

Spirometry is a simple non-invasive method that evaluates only volumes above the residual volume and flow volume rates (21). Spirometric values were measured using a spirometer (P.K. Morgan Ltd., UK). Spirometric values were obtained with the patients seated, with clipped nose and with no restrictions to chest expansion, such as fitted or tight clothes, and no orthodontic braces. To obtain accurate results, cases were encouraged to attempt the test a maximum of five times and the maximal result was recorded. All measurements were completed during a maximum 2-minute interval.

The respiratory test was carried out by the same operator, after careful training. The operator and gave plenty of encouragement to the children to perform the tests.

Children were assessed twice; 1 week before and 40 days after surgery in the same clinic and by the same operator. As well as spirometric parameters, snoring and duration of disease and its associated symptoms such as enuresis, open mouth breathing, and hyponasal sound were evaluated through questionnaires pre- and post-operatively. The severity of snoring was evaluated using a visual analog scale (VAS). The significance of changes in respiratory indices and the correlation between spirometric values and symptom scores were analyzed by Student's and McNemar's tests.

The protocol was approved by the Ethics and Research Committee of Isfahan University of Medical Sciences. Parental consent was also signed before evaluation for each patient.

## Results

The sample group consisted of 42 children (19 female and 23 male) aged between 5 and 11 years (mean, 6.9±1.7 years). Two female children were excluded from the study because relocation meant that they did not perform the second procedure. Therefore, the analysis was performed on 40 cases. All patients presented with nasopharyngeal obstruction (A/N ratio> 0.70). Twenty-nine patients exhibited grade III and the others had the grade IV enlarged tonsils.Table 1 shows the improvement in forced vital capacity (FVC), peak expiratory flow (PEF), maximal expiratory flow at 25% of FVC (MEF_25_), and mid expiratory forced expiratory flow (FEF_25-75_) 40 days after surgery as compared with preoperative measures (P=0.02).

**Table 1:** Mean Pre-adenotonsillectomy and Post-adenotonsillectomy spirometric parameters

Spirometric Parameters	Pre-adenotonsillectomy (Mean±SD)	Post-adenotonsillectomy (Mean±SD)	P-value
**FEV1**	1.21±0.23	1.23±0.21	0.41
**FVC**	1.28±0.26	1.33±0.24	0.04
**FEV1/FVC**	95±4	93±5	0.07
**FEF **_25-75_	1.81±0.5	1.91±0.48	0.02
**PEF**	2.74±0.65	2.84±0.51	0.02
**MEF **_25_	1.09±0.36	1.21±0.34	0.02
**MEF**_50_	2.13±0.47	2.09±0.50	0.384
**MEF **_75_	2.68±0.57	2.72±0.45	0.498

SD: standard deviation, FEV_1_: forced expiratory volume during the first second of expiration, FVC: forced vital capacity, FEV1/FVC: forced expiratory volume during the first second of expiration to forced vital capacity, FEF _25-75_: midexpiratory forced expiratory flow, MEF _25_: maximal expiratory flow at 25% of FVC, MEF_50_: maximal expiratory flow at 50% of FVC_, _MEF _75_: maximal expiratory flow at 75% of FVC.

We found no statistically significant differences in forced expiratory volume during the first second of expiration (FEV_1_), forced expiratory volume during the first second to forced vital capacity (FEV_1_/FVC) ratio, maximal expiratory flow at 50% of FVC (MEF_50_), or maximal expiratory flow at 75% of FVC (MEF_75_) for the same period (P>0.05). 

Snoring score was obtained using a VAS. The mean VAS score decreased from 62.9±30.9 pre-operatively to 2.9±4.6 post-operatively (P<0.001). 

**Fig 1:**
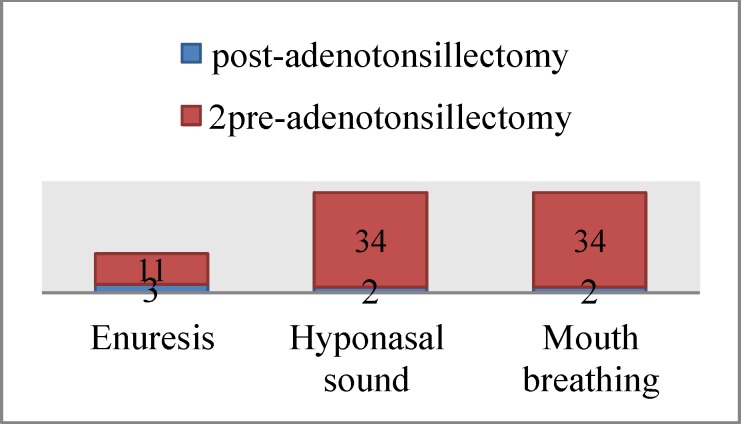
frequency of enuresis, Hyponasal sound and mouth breathing pre and post adenotonsillectomy

Before adenotonsillectomy, 11 patients (27.5%) reported enuresis, 34 patients (85%) experienced hyponasal sound, and 34 patients (85%) reported mouth breathing. Forty days after the operation, these numbers changed to three (7.5%), two (5%), and two (5%), respectively (Fig.1).

There was no correlation between changes in spirometric parameters and snoring and the changes assessed in terms of VAS (Table.2). Changes in FEF_25–75 _were correlated with changes in other spirometric parameters (P<0.05, r>0); i.e. improvement in each of the spirometric parameters was concomitant with improvement in FEF_25–75_ and vice versa.

**Table 2:** Pearson's correlation coefficient in changed spirometric parameters and snoring and its changes

Changed spirometric parameters	VAS of snoring		VAS of changes in snoring	
	r	P-value	r	P-value
**FEV1**	-0.15	0.17	0.12	0.23
**FVC**	-0.14	0.19	0.12	0.23
**FEV1/FVC**	0.04	0.40	0.02	0.44
**FEF **_25-75_	-0.98	0.27	0.08	0.32
**PEF**	0.09	0.29	-0.11	0.25
**MEF **_25_	-0.19	0.11	0.18	0.14
**MEF**_50_	-0.16	0.16	0.12	0.23
**MEF **_75_	-0.06	0.35	0.03	0.43

VAS: visual analogue scale, r: Pearson'scorrelation coefficient, FEV_1_: forced expiratory volume during the first second of expiration, FVC: forced vital capacity, FEV1/FVC: forced expiratory volume during the first second of expiration to forced vital capacity, FEF _25-75_: midexpiratory forced expiratory flow, MEF _25_: maximal expiratory flow at 25% of FVC, MEF_50_: maximal expiratory flow at 50% of FVC_, _MEF _75_: maximal expiratory flow at 75% of FVC.

## Discussion

Upper airway obstruction can be evaluated by a non-invasive technique such as PFT (spirometry) which helps to detect those obstructive changes that might not be clinically evident (22).

In the present study, we found improvement in FVC, PEF, MEF_25_ and FEF_25–75_ 40 days after surgery in children with enlarged tonsils as compared with baseline measures. This finding is in contrast with a number of other studies (12,15,23). For example, Yadav et al. reported significant improvement in FIF_50_ and FEF_50_/FIF_50, _FEV_1_/PEFR, and FEV_1_/FEV_0.5_ ratios following surgery (22). Similar to our findings, Kavukcu et al. reported an increase in FVC, PEF, FEV_1_, MEF_25_, FEF, and FEF_75_ after adenotonsillectomy (18).

Some authors believe that the lower airway shows obstructive changes in the children with ATH. They believe that disorders of the upper and lower airways commonly coexist, as they present a similar histology that may be considered in the context of united airways (24,25).

On the other hand, obstruction of the upper airway can cause alterations in other parts of the respiratory system, as well as in the amount of muscle stretch and thoracic movement (26). Children with ATH tend to have trouble with nasal breathing, and therefore prefer to breathe through the mouth (12). It has been also assumed that less nasal humidification and warming due to mouth breathing can lead to alterations in the diffusion and viscosity of the surfactant which may be a powerful stimulus for bronchiolar obstruction (27). Chronic mouth breathing may also impose some undesired physical effects on respiratory function, by deforming the craniofacial skeleton, oral cavity and posture, and also on psychological characteristics (28).

Moreover, relative hypoxemia and hypercarbia are seen in many patients with ATH (11). The decrease in both maximum breathing capacity and respiratory response to CO_2 _(29) may be responsible for alterations in pulmonary function, as seen in the functional indices of spirometry. Furthermore, tonsils and adenoids are the principal parts of Waldeyer's ring which its basic role is antibody synthesis. Chronic ATH is recognized by chronic inflammation and hypoxia/reoxygenization episodes. This may lead to synthesis of inflammatory cytokines, and free nitrogen and oxygen radicals (30, 31) which may be responsible for impaired pulmonary function.

Finally, as described by other researchers, inflammation of the nasal and oropharyngeal mucosa is a common finding, as in adults with sleep-disordered breathing (SDB) (32). C-reactive protein, a systemic indicator for inflammation, has recently been found to be elevated in the serum of children with ATH (33). Additionally, expression of glucocorticoid receptors in the upper airway lymphoid tissues and expression of inflammatory mediators and leukotriene receptors were increased in children with SDB (34-36).

We suggest that the increased inflammatory conditions have some effect on the upper and lower airway in these children. The mechanisms which promote this inflammatory reaction are probably related, at least partially, to the vibratory mechanical injury associated with snoring (37). However, we found no correlation between clinical findings such as snoring and changes in spirometric parameters.

In our study, we observed changes in a number of obstructive spirometric indices. Our study was designed to be as simple as possible with a low cost. This simplicity facilitates the reproducibility of similar studies in routine medical practice. On the other hand, it is often difficult to obtain accurate measurements through spirometry because of poor cooperation in performing the test in children (18). PFT could reveal the obstructive influence of ATH and could be valuable in surgical indications of ATH due to recurrent infections in children (18,22). It could also be beneficial in convincing hesitant parents of the importance of a timely decision with respect to adenotonsillectomy.

## Conclusion

Although adenotonsillectomy might have a positive effect on the function of the lower respiratory tract, we found no significant correlation between changes in spirometric parameters and snoring. However, performing a spirometric examination in children with ATH may be beneficial for assessing the pulmonary function of these patients.

It seems that PFT can reveal the obstructive effect of ATH whenever slight clinical or radiological obstructive signs are present, and might be valuable in surgical indications of ATH due to recurrent tonsillar infections in children with a mildly obstructed airway. Further studies are needed to explore the precise etiology of these changes.
